# Ethno-medicinal study of plants used for treatment of human and livestock ailments by traditional healers in South Omo, Southern Ethiopia

**DOI:** 10.1186/1746-4269-9-32

**Published:** 2013-05-16

**Authors:** Ketema Tolossa, Etana Debela, Spiridoula Athanasiadou, Adugna Tolera, Gebeyehu Ganga, Jos GM Houdijk

**Affiliations:** 1 Department of Chemistry, College of Natural and Computational Science, Hawassa University, P.O. Box 05, Hawassa, Ethiopia; 2 School of Veterinary Medicine, Hawassa University, P.O. Box 05, Hawassa, Ethiopia; 3 Disease Systems, SRUC, Edinburgh, United Kingdom; 4 School of Animal and Range Science, College of Agriculture, Hawassa University, P.O. Box 222, Hawassa, Ethiopia; 5 Livestock Research Directorate, South Agriculture Research Institute (SARI), P.O. Box 06, Hawassa, Ethiopia

**Keywords:** Ethnomedicine, Traditional healers, Medicinal plants, South Omo, Ailments, Informants consensus factor

## Abstract

**Background:**

Plants have traditionally been used for treatment of human and livestock ailments in Ethiopia by different ethnic and social groups. However, this valuable source of knowledge is not adequately documented, which impedes their widespread use, evaluation and validation. Here, we recorded indigenous knowledge and standard practices for human and livestock disease control, of three ethnic groups (Aari, Maale and Bena-Tsemay) in South Omo Zone of Southern Nations, Nationalities and Peoples Regional State, Ethiopia.

**Methods:**

A cross-sectional study was carried out using a semi-structured questionnaire to document knowledge of 50 traditional healers (40 male and 10 female) in medicinal plant use for treatment of human and livestock ailments. Descriptive statistics were used to analyze and summarize the ethno-botanical data.

**Results:**

Ninety-one plants, with claimed medicinal properties against a total of 34 human and livestock ailments, were reported and botanically identified as belonging to 57 genera and 33 plant families. Most of the plant species reported belonged to one of seven major families: *Lamiaceae*, *Solanaceae*, *Menispermiaceae, Fabaceae*, *Asteraceae*, *Plumbaginaceae* and *Geraniaceae*. Woody plants (shrubs 21% and trees 29%) were the major growth form used, whilst roots (40%) and leaves (35%) were the major plant parts used in the study areas. Healers mostly practice oral administration of plant preparations (65%). Multiple medicinal plants were cited against particular ailments, and mixing of two or more different medicinal plants (14.3%) against a single ailment was also commonly reported.

**Conclusion:**

This study showed that traditional medicine, mainly involving the use of medicinal plants, is playing a significant role in meeting the primary healthcare needs of the three ethnic groups. Acceptance of traditional medicine and limited access to modern healthcare facilities could be considered as the main factors for the continuation of the practice. Documented knowledge of the traditional healers can be used to support the country’s human and livestock health care system and improve lives and livelihoods. Information generated will be used in future studies to validate bioactivity of selected medicinal plants used by traditional healers, so to increase their acceptability in health care systems both nationally and internationally.

## Background

Knowledge can arise from scientific or traditional sources [1]. Traditional knowledge has been described as a cumulative body of knowledge, practice and belief, evolving through adaptive processes and handed over through generations by cultural transmission [2]. Traditional medicine is used throughout the world as it is heavily dependent on locally available plant species and plant-based products and capitalizes on traditional wisdom-repository of knowledge [3]. The wide spread use of traditional medicine could be attributed to cultural acceptability, economic affordability and efficacy against certain type of diseases as compared to modern medicines. Thus, different local communities in countries across the world have indigenous experience in various medicinal plants where they use their perceptions and experience to categorize plants and plant parts to be used when dealing with different ailments [4].

Plants have played a central part in combating many ailments in human and livestock in many indigenous communities, including Africa [5]. Traditional healers, and particularly medicinal plant herbalists, in Africa have a detailed knowledge-base of traditional medicine [6,7], which is transferred orally from one generation to the next through professional healers, knowledgeable elders and/or ordinary people [8].

In Ethiopia, traditional medicine has played a significant role in treating health problems in both livestock and humans [9-12]. Knowledge of medicinal plants of Ethiopia and of their uses provides vital contribution to human and livestock health care needs throughout the country [13]. The plant- based human and livestock health care persists and remains as the main alternative treatment for different ailments in Ethiopia, largely due to shortage of pharmaceutical products, prohibitive distance of the health service stations, unaffordable prices by small holder farmers and pastoralists for conventional drugs, emergence and re-emergence of certain diseases and appearance of drug resistant microbes and/or helminthes [14].

Much of the Ethiopian South Omo population is made up of nomadic pastoralists who depend upon livestock as their main source of livelihood [15]. The traditional medicinal plant lore and potentials of the three ethnic groups have not been investigated to a conspicuous level. Similar to many other rural communities in Ethiopia, the use of traditional medicinal plants plays a vital role in human and livestock health care systems in the pastoral and agro-pastoral communities of the study areas. The three ethnic groups (Aari, Maale and Bena-Tsemay) in South Omo are expected to be custodians of valuable indigenous knowledge on the use of their traditional medicinal plants, which they use for treating of human and livestock ailments. Currently, access to modern health services for both human and livestock is very limited and/or non-existent for some community members of the study areas. This study is basically focusing on a remote and pastoralist areas where the accessibility, affordability and cultural acceptability of the use of medicinal plants for treatment of human and livestock ailments is very significant. The dependence of the plant-based health care system could partly be attributed to underdeveloped infrastructures and modern medical health care system in the general area. Unless the plants are conserved and the ethno-medicinal knowledge is documented, there is a danger that both the valuable medicinal plants and the associated indigenous knowledge of the ethnic groups could vanish forever due to lack of documentation [6] and loss of valuable medicinal plants due to population pressure, agricultural expansion and deforestation [16], as well as due to drought, urbanization and acculturation [17]. Furthermore, pastoral and agro-pastoral communities of these ethnic groups have remained ethno-medicinally unexplored and there is no comprehensive account of the medicinal plant-based practices. Therefore, the objective of this study was to document the indigenous knowledge and practices of the healers in the study areas (the three ethnic societies in the South Omo zone of the Southern Nations, Nationalities and Peoples Region (SNNPR) of Ethiopia) on medicinal plants for human and livestock disease control. Below, we describe the study area, how informants were selected, the type of information we have gathered, and the use of the informant consensus factor to synthesize the information obtained, followed by presenting our findings and discussing them in context of existing literature.

## Materials and methods

### Description of the study area and the people

**Figure 1:**
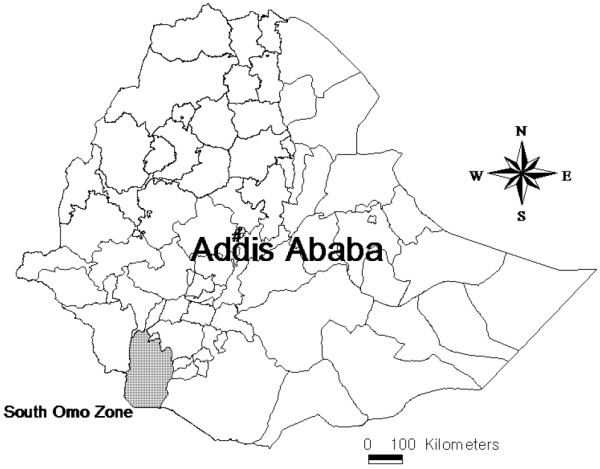
Map of South Omo zone indicated by the shaded region.

The study was conducted in selected areas of the South Omo zone, inhabited by three ethnic groups (Aari, Maale and Bena-Tsemay). The administrative zone is bordered on the south by Kenya, on the west by Bench Maji, on the northwest by Kefa-Sheka, on the north by North Omo, on the northeast by the Derashe and Konso Special Woredas, and on the east by the Oromia Regional State (Figure 1).

The annual average temperature of the area is 28°C with an annual average rainfall of 1190 mm [18]. The 2007 census data revealed that the zone has a total population of 573,435 people (286,607 male and 286,828 female) of which 4.45% are pastoralists and semi-pastoralists. Their culture places highest value on cattle, with relatively less on mixed farming (Central Statistics Agency, 2007). Among the six largest ethnic groups living in the area, Aari, Maale and Hamer-Bena (Bena-Tsemay) comprise 42.9%, 13.5% and 12.9%, respectively. Aari, Maale and Hamer are the major languages spoken by 43.3%, 13.7% and 13% of the people in the area, respectively [19].

### Selection of informants

A total of 50 traditional healers, i.e. 24 from the Aari (20 males and 4 females), 16 from the Maale (13 males and 3 females) and 10 from Bena-Tsemay (7 males and 3 females) ethnic groups of different ages (32–81 years) were selected with the help of local elders, agricultural and health extension workers and administrative personnel, and interviewed as key informants. The selected healers were well-known in the community due to their long practice in providing services related to traditional health care to the community. Prior to the interview process, discussion was held with the informants through assistance of local elders to elaborate the objective of the study. This was done to clarify the purpose and build confidence of the respondents to provide reliable information without suspicion. After the discussion, 6 potential informants (2 from Aari, 1 from Maale and 3 from Bena-Tsemay) showed unwillingness to share their medicinal plant knowledge and withdrew from the study. The 50 healers that participated in the study were asked to provide information on plant(s) use against any kind of illness in humans and livestock, and in particular the type of plant (e.g. trees, shrubs, herbs, climbers or others) and the parts used (e.g. roots, leaves, seeds, flowers, stems or others), the methods of remedy preparation (e.g. concoction, filtrate, paste on, smoke bath, pounded or others), the routes of administration (e.g. oral, topical, smoke bath, nasal or others) and the dosage. Specimen of the reported medicinal plants were collected during the interview from the field, coded and sent to the National Herbarium of Addis Ababa University for botanical identification and archiving.

### Data collection and analysis

Descriptive statistics were used to analyze and summarize the ethno-botanical data. Based on the information obtained from the informants, the ailments reported were grouped into a total of 12 categories (Table 1). To estimate medicinal plant use variability and to assist prioritizing medicinal plants for further studies, the informant consensus factor (ICF) was calculated [20]. The ICF is calculated using the number of use citations (Nuc) in each category and the number of plant species (Ns) cited through the following formula:

$$\mathrm{ICF}=\frac{\mathrm{Nuc}-\mathrm{Ns}}{\mathrm{Nuc}-1}$$

Thus, ICF values range from 0 to 1, with high values (i.e. close to 1) indicating that relatively few plants are used by a large proportion of informants, while low values (<0.5) indicate that informants do not agree on the plant species to be used to treat a category of ailments.

**Table 1:** Informant consensus factor (ICF) values of use category of multiple plants claimed as having medicinal values by informants of the three ethnic groups from South Omo zone, southern Ethiopia

**Use category**	**Plant species**	Number of use citation^*^	**% of all citations**	**ICF value**
Anthrax	*Dombeya* spp. (7), *Achyrospremum schimperi* (6), *Tragia doryodes* (5), *Geranium aculeolatum* (8)	26	6.5	0.88
Poisonous plants	*Solanum* spp. (5), *Oxalis corniculata* (7)	12	2.9	0.91
Skin infections and external parasites	*Solanum incanum* (6), *Desmodium dichotomum (*7), *Laggera tomentosa* (8), *Geranium arabicum* (6), *Nicotiana tabacum* (6), *Premna schimperi* (9), *Calpurnia aurea* (8)	50	12.4	0.88
Pain related illnesses	*Senna* spp. (4), *Tagetes* spp*.* (9), *Monsonia parvifolia* (8), *Plumbago caerulea* (4), *Desmodium delotum* (3)	28	6.9	0.85
Malaria and anemia like syndrome with jaundice	*Carphalea glaucescens* (5), *Cissampeolos mucronata* (6), *Indigofera arrecta* (6), *Cissampelos* spp. (2), *Lantana trifolia* (4), *Luecas stachydiformis* (2), *Premna oligotricha* (5), *Droguetia iners* (6), *Zornia glochidiata* (3), *Galinsoga parviflora* (3)	40	9.9	0.77
Abdominal/stomach disorders and internal parasites	*Pycnostachys meyeri* (5), *Zornia apiculata* (4), *Achyranthes aspera* (4), *Stereospermum kunthianum* (7), *Cissampelos pariera* (6), *Cissampelos capensis* (3), *Lagenaria siceraria* (3), *Momordica charantia* (2), *Tagetes minuta* (6), *Premna* spp. (5), *Salvia acuminata* (4), *Echinacea* spp. (2), *Pelargonium alchemilloides* (5), *Orthosiphon surmentosus* (6), *Mucuna melanocarpa* (8), *Sida* spp. (5), *Chasmanthera welwitschii* (2), *Rytigynia* spp. (5), *Zornia latifolia* (3), *Centella asiatica* (3), *Ipomoea eriocarpa* (7), *Chlaenandra ovata* (2)	97	24.1	0.78
Snake bite/poisoning	*Alonsoa acutifolia* (5), *Plumbago pulchella* (6), *Opericulicarya gummifera* (6), *Plectranthus globosus* (6), *Barthlottia madagascariensis* (3), *Ludwigia abyssinica* (6), *Claoxylopsis andapensis* (8), *Carissa carandas* (5), *Hyparrhenia hirta* (4), *Verbena officinalis* (5)	54	13.4	0.83
Mich and Megagna^**^	*Dobera* spp. (6), *Droguetia debilis* (5), *Justica dianthera* (4)	15	3.7	0.86
Coughing in equines and ruminants	*Justicia diffusa* (5), *Solanum bellum* (5), *Datura stramonium* (4), *Vaccaria hispanica* (6), *Ozoroa insignis* (6)	26	6.5	0.84
Removal of retained placenta	*Solanum acaule* (2), *Solanum acuminatum* (3), *Dovyalis* spp. (5), *Galinsoga quadriradiata* (6), *Colocasia esculenta* (4), *Plumbago zeylanica* (6), *Momordica* spp*.* (2)	28	6.9	0.78
Evil eye	*Drymaria* spp. (4), *Plectranthus glabriflorus* (3), *Cissus quadrangularis* (4), C*ryptocarpus* spp*.* (3), *Colignonia ovalifolia* (4), *Achyrospermum africanum* (5), *Drymaria cordata (*3), *Plumbago auriculata* (4), *Chelonopsis moschata* (2), *Withania somnifera* (3), *Plumbago* spp. (2)	37	9.2	0.72
Black leg	*Momordica foetida* (7), *Pentas suswaensis* (8), *Chasmanthera dependens* (3)	18	4.5	0.88
Rabies	*Caylusea abyssinica* (3)	3	0.7	1.00
Improve milk production in cows	*Indigofera trita* (6)	6	1.4	1.00

^*^ Numbers in parenthesis indicate the number of citation of that plant by informants (traditional healers and community members) against a particular ailment category.

^**^ Ailment characterized with fever, head ache and sweating.

## Results and discussion

### Knowledge of informants on medicinal plants

**Figure 2:**
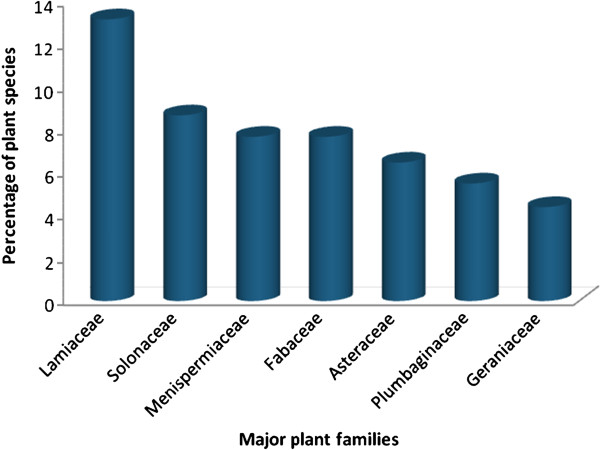
The percentage of plant species distributed over seven major families.

Indigenous people of different localities have their own specific knowledge on plant use, management and conservation [21]. Medicinal plants represent a significant contribution to human and livestock health and it has been suggested that their use is one of the most significant ways in which humans directly reap the benefits provided from biodiversity [22,23]. During the field survey in our study areas, informants reported ethno-medicinal data of 91 species of plants distributed across 33 families and 57 genera as having medicinal properties against 34 ailments (12 in humans, 11 in livestock and 11 in both human and livestock). The 91 plant species that are used by traditional healers among the three ethnic groups interviewed were identified and documented. Among the medicinal plants identified most of them belong to the seven families as shown in Figure 2. The plant family *Lamiaceae* was most frequently represented amongst the documented useful species, with a total of 12 species out of the 91 plants identified, followed by *Solanaceae* with a total of 8 species and *Menispermiaceae*, and *Fabaceae* with total of 7 species each, and others constitute one up to six plant species per family.

The informants cited 32 (35.2%), 35 (38.5%) and 24 (26.4%) plants as having medicinal properties against ailments of livestock, humans or both livestock and humans, respectively (Table 2). The informants also reported multiple plant remedies against certain ailments, such as snake bite/poisoning for both humans and livestock. Depending upon the type of illness, the use of two or more parts of medicinal plants was also reported by some healers as common practice. For example, of the total 32 medicinal plants purely claimed for livestock illnesses, eight were used in two-plant combination preparations, and these target three different ailments, i.e. to treat epizootic lymphangitis, removal of fetal membrane, and anemia with jaundice (Table 2).

**Table 2:** Medicinal plants, with family, scientific and local name, for selected ailments of human and/or veterinary importance, with parts used and preparations, as claimed by informants of the three ethnic groups from South Omo zone, Southern Ethiopia

**Family**	**Scientific name**	Local name^*^	**Voucher number**	**Use(s)**	**Parts used and preparation**	**Importance**
Amaranthaceae	*Achyranthes aspera* Linn.	Busino (M)	KTG28	Abdominal pain and tonsillitis	Root chopped and mixed with water and taken orally. Leaf chewed and the extract kept near the inflammation area	Human
Lamiaceae	*Achyrospermum africanum* Hook.f. ex Baker	Kebit buda (A)	KTG54	Evil eye	Leaf and root chopped and soaked with water	Human
	*Achyrospermum schimperi* (Hochst. ex Briq.) Perkins	Abasanga medihanit (A,Amh)	KTG65	Anthrax	Leaf and flower chopped and soaked with hot water and drenched	Veterinary
Scrophulariaceae	*Alonsoa acutifolia* Ruiz & Pav.	Shosha tesha (M)	KTG66	Snake bite/poison	Root chopped and mixed with *Plectranthus glandulosus* in water and the filtrate drenched	Human and Veterinary
	*Barthlottia madagascariensis* E.Fisch.	Unkown (A)	KTG18	Snake bite/poison	Concoction	Human and Veterinary
Fabaceae	*Calpurnia aurea* (Aiton)Benth.	Kaino(M)	KTG91	Flea and louse infestation	Freshly chopped or dried and ground leaf mixed with water and applied to the flea and louse infested areas	Veterinary
Apocynaceae	*Carissa carandas* L.	Goiti(B),ebab medihanit (Amh)	KTG45	Snake bite/poison	The leaf chopped, mixed with water taken orally	Human and Veterinary
Rubiaceae	*Carphalea glaucescens* (Hiern) Verdc	Wariamo (M)	KTG31	Anaemia (known as Airo)	Leaf powdered mixed with *Ipomoea kiwuensis* smoked for three days	Human
Recedaceae	*Caylusea abyssinica* (fresen.) Fisch. & Mey	Giesilla (M)	KTG64	Rabies (effective even when clinical signs are present)	Root chopped and mixed with cold water and drenched	Human and Veterinary
Apiaceae	*Centella asiatica* - (L.)Urb.	Busino (M)	KTG87	Abdominal ache	Root dried, ground and mixed with cold water when needed (on cup or glass full)	Human
Menispermaceae	*Chasmanthera dependens* (Hochst)	Moshito (M)	KTG33	Black leg	Root bark and leaf dried and ground and given to emaciate calf as much as possible	Veterinary
	*Chasmanthera welwitschii* Troupin	Heilho (M)	KTG27	Antiamoeba	Root bark and leaf dried and ground and given to emaciate calf as much as possible	Veterinary
Lamiaceae	*Chelonopsis moschata* Miq.	Kebit buda (A)	KTG47	Evil eye	Leaf and root chopped and soaked with water	Human
Menispermaceae	*Chlaenandra ovata*. Miq.	Eincht (A)	KTG73	Abdominal ache	Root chopped and mixed with water and drenched	Human
	*Cissampelos capensis* L.f*.*	Wontin kanna (A)	KTG76	Abdominal cramp	Root chopped, powdered, soaked with water, filtered and drenched	Human
	*Cissampelos mucronata* A.Rich.	Kawuro (M)	KTG37	Anaemia with jaundice	Leaf collected, dried, ground and mixed with hot water and two spoonful taken at once	Human
	*Cissampelos pareira* L*.*	Shelindo (M)	KTG70	Broad spectrum anti- helminthiasis	Root ground mixed with large amount of water and drenched. It causes fever diarrhea then animals are cured	Veterinary
	*Cissampelos* spp.	Kawto (M)	KTG32	Anaemia like syndrome with jaundice	Leaf collected, dried, ground with local mill and mixed with hot water and two spoonful taken at once (bitter)	Human
Vitaceae	*Cissus quadrangularis* L.	Bararo (M)	KTG20	Evil eye	Tied under belly	Veterinary
Euphorbiaceae	*Claoxylopsis andapensis* Radcl.-Sm.	Dorba (A)	KTG43	Snake bite/poison	Bark and leaf chopped, soaked in water and drenched	Human
Nyctaginaceae	*Colignonia ovalifolia*Heimerl	Afesha (A)	KTG41	Evil eye	Leaf squeezed and inhaled	Human
Araceae	*Colocasia esculenta* (L.) Schott	Haleko (A,M,BT)	KTG77	To detach retained fetal membrane	Root dried, ground and mixed with powdered root of *Momordica* spp. and all soaked in warm water and one cupful drenched	Veterinary
Nyctaginaceae	*Cryptocarpus spp*.	Afei tesha (M)	KTG23	Evil eye	Root chopped and mixed with cold/hot water	Human
Solanaceae	*Datura stramonium* L.	Onidod (A)	KTG05	Coughing (for horses, mules and donkeys)	Leaf chopped and mixed with cold water and drenched via nose	Veterinary
Papilionaceae	*Desmodium dichotomum* (Willd.) DC.	Muasii (A)	KTG07	Epizootic lymphangitis (tushita)	Root chopped, mixed with cold water and drenched via nose	Veterinary
	*Desmodium delotum* J.F. Macbr.	Not known (A)	KTG42	Eye illness	Leaf apex chopped, soaked in water, applied to sick eye	Human and Veterinary
Salvadoraceae	*Dobera* spp.	Mitch medihanit (A, Am)	KTG89	Mitch	Leaf boiled with water and inhaled	Human
Sterculiaceae	*Dombeya* spp.	Bata (A)	KTG15	Anthrax	The leaf is chopped and mixed with *Tragia doryodes* and the filtrate taken orally	Human and Veterinary
Flacortiaceae	*Dovyalis* spp.	Mukale (M)	KTG85	To detach retained placenta	Leaf chopped and mixed with hot water and given as ad libtum	Veterinary
Urticaceae	*Droguetia debilis* Rendle	Megagna medanit (Amh)	KTG52	Megagna	Leaf apex chopped and pasted on the pain area	Human
	*Droguetia iners* (Forssk.) Schweinf.	Yewoba medihanit (A)	KTG61	Malaria	Leaf chopped and mixed with *Premna oligotricha* and boiled together one glassful drenched	Human
Caryophyllaceae	*Drymaria cordata* (L.) Willd. ex Schult.	Yebuda medihanit (A, Amh)	KTG59	Evil eye in animals	Leaf and root chopped mixed in water and the filtrate is sprayed on animal body and the sediments are drenched	Human and Veterinary
	*Drymaria* spp*.*	Unknown (A)	KTG17	Evil eye	The leaf is chopped and mixed with water and the filtrate taken orally	Human
Asteraceae	*Echinacea* spp.	Unkown	KTG53	Diarrhoea alone	Root chopped and soaked in warm water taken orally	Human and Veterinary
	*Galinsoga parviflora* Cav.	Midirberbere, (Amh)	KTG14	Anaemia with jaundice	The flower is chopped and mixed with *Monosonia longipes* and warmed on and applied of gum of achy tooth	Human
	*Galinsoga quadriradiata* Cav.	Mukala (M)	KTG74	To detach retained fetal membrane and/or placenta	Leaf chopped and mixed with *Plumbango zeylanica* and then drenched	Veterinary
Geraniaceae	*Geranium aculeolatum* Oliv.	Abasanga medihanit (A, AM)	KTG72	Anthrax	Leaf chopped and rubbed on wounded part	Veterinary
	*Geranium arabicum* Forssk.	Tushita (A)	KTG26	Epizootic lymphangitis	Root chopped and mixed with chopped root of *Laggera tomentosa* and one bear bottle drenched through left nose of horse.	Veterinary
Poaceae	*Hyparrhenia hirta* (L.) Stapf	Goiti ebab” medihanit (B)	KTG46	Snake bite/poison	Plant material chopped and soaked in hot water and the filtrate drenched	Human and Veterinary
Fabaceae	*Indigofera arrecta* A.Rich.	Wareami (A)	KTG80	Anaemia with jaundice	Leaf dried and smoked to patients	Human
	*Indigofera trita* L.f.	Wusis (A)	KTG01	To improve milk production of cows	Root chopped, mixed with water and drenched	Veterinary
Convolvulaceae	*Ipomoea eriocarpa* R. Br.	Choko (M)	KTG40	Endoparasite	Root chopped and mixed with water, then the filtrate is drenched and rest sediments are poured on the wound part	Veterinary
Acanthaceae	*Justica dianthera* Vell.	Mitch(A, Amh)	KTG51	Mitch	The leaf apex boiled with water and the vapor inhaled and/or the filtrate drenched	Human
	*Justicia diffusa* Willd.	Makaiso (A)	KTG04	For coughing of equines	Leaf chopped and mixed with cold water and drenched via nose	Veterinary
Cucurbitaceae	*Lagenaria siceraria* (Molina) Standl.	Busino (M)	KTG35	Diarrhoea and vomiting	Leaf chopped and ground and the drench the filtrate	Human
Asteraceae	*Laggera tomentosa* Schultz Bip.	Tushita (A)	KTG71	Epizootic lymphangitis	Root chopped and mixed with chopped root of *Geranium arabicum* and one bear bottle drenched through horses nose	Veterinary
Verbenaceae	*Lantana Trifolia* L.	Yewoba medihanit (A)	KTG55	Malaria (shivering type, vivax)	Root chopped and soaked with water and mixed with local alcoholic drink (Areke)	Human
Lamiaceae	*Leucas stachydiformis* (Benth.) Hochst. ex Briq.	Businae (M)	KTG19	Anaemia with jaundice	Leaf and bark chopped and drench the filtrate or inhalation	Human
Onagraceae	*Ludwigia abyssinica* A.Rich.	Yechira ebab medihanit (Amh)	KTG44	Snake bite/poison	Stem and root mixed with other plants and applied orally	Human
Cucurbitaceae	*Momordica foetida* Schumach.	Chekko (M,B)	KTG03	Black leg	Root chopped, soaked in water for half a day and a filtrate is drenched	Veterinary
	*Momordica charantia* L.	Unknown (A)	KTG90	Diarrhea and vomiting	Leaf and root ground well and mixed with milk and taken orally	Human
	*Momordica* spp.	Kill (M)	KTG79	To detach fetal membrane	Root dried, ground and mixed with *Colocasia esculenta*, all soaked in warm water and one cupful filtrate drenched	Veterinary
Geraniaceae	*Monsonia parvifolia* Schinz	Not known (A)	KTG58	Tooth ache	Seed and leaf crushed and mixed with salt and *Galinsoga parvifolia*; made hot on fire with “enset leaf” and applied on gum.	Human
Papilionaceae	*Mucuna melanocarpa* Hochst	Salabano (M)	KTG86	For calf Ascariasis	Leaf ground and mixed with water and drenched that induces diarrhea	Veterinary
Solanaceae	*Nicotiana tabacum* L.	Bangiso(M)	KTG78	Tick infestation	Root chopped and mixed with water and dressed to the tick infested area on cow and calf	Veterinary
Anacardiaceae	*Operculicarya gummifera* (Sprague) Capuron	Dorba (M)	KTG38	Snake bite/poison	Orally taken butt its preparation is not specified due to unwillingness of the respondent	Human and Veterinary
Lamiaceae	*Orthosiphon sarmentosus* A.J. Paton & Hedge	Zititu (A)	KTG67	Ascariasis	Leaf chopped, soaked in water and a glass full filtrate drunken	Human
Oxalidaceae	*Oxalis corniculata* L*.*	Dani (M)	KTG02	Toxic	Root chopped, cooked for two days and more and the paste rubbed on arrow tip to hunt wild animals	Human and veterinary
Anacardiaceae	*Ozoroa insignis* Delile	Bussa (M)	KTG39	For coughing of equines	Bark dried, powdered and mixed with cold water and the filtrate drenched	Veterinary
Geraniaceae	*Pelargonium alchemilloides* (L.) Aiton	Unkown	KTG13	Constipation	Root chopped and soaked in warm water taken orally	Human
Rubiaceae	*Pentas suswaensis* Verdc.	Haromato (M)	KTG63	Aba gorba “Black leg”	Leaf chopped and mixed with boiled water and the filtrate is drenched	Veterinary
Lamiaceae	*Plectranthus glabriflorus* P.I.Forst.	Gullo/Karika (A)	KTG09	Evil eye	Leaf soaked in hot water and drunken	Human
	*Plectranthus globosus* Ryding	Chambuase (M)	KTG60	Snake bite/poison	Leaf chopped mixed with *Alectra sessiliflora* mixed in cold water and taken orally	Human
Plumbaginaceae	*Plumbago auriculata* Lam.	Masilok (M)	KTG81	For animal evil spirit	Leaf chopped and soaked in water and the filtrated drenched and the remaining sediments pasted on the body	Human and Veterinary
	*Plumbago caerulea* Kunth	Wugat medihanit (Amh)	KTG68	Back and side pain	Root and seed are chopped and mixed with hot water and onion	Human
	*Plumbago pulchella* Boiss.	Not known (A)	KTG12	Snake bite/poison	Fresh leaf chopped and mixed with cold water	Human and Veterinary
	*Plumbago* spp.	Misirich (M)	KTG49	Evil eye in animals	Leaf chopped and soaked in water and the filtrated drenched and the remaining sediments pasted on the body	Veterinary
	*Plumbago zeylanica* L*.*	Telba (M)	KTG75	To detach retained fetal membrane	Seed ground by traditional mortar mixed with *Galinsoga Parviflora* and boiled with water and drenched	Veterinary
Lamiaceae	*Premna oligotricha* Baker	Yewoba medihanit (A)	KTG34	Malaria (non-shivering type, falciparium)	Leaf collected and ground and mixed with water	Human
	*Premna schimperi* Engl.	Bangizo(M)	KTG21	Dermatophilous and mite infestation	Root chopped and soaked in warm water over night and filtrate applied topically to treat dermatophytes and tick mite infestations	Veterinary
	*Premna* spp.	Anchiphi (M)	KTG57	Diarrhea in calf	Leaf powdered and mixed with water and the filtrate drenched	Veterinary
	*Pycnostachys meyeri* Gürke ex Engl.	Unkown (A)	KTG83	Abdominal pain (children)	Fresh root chopped and mixed with cold water and drenched	Human
Rubiaceae	*Rytigynia* spp.	Golodo (M)	KTG30	Typhoid (Micho)	Leaf chopped and mixed with water and taken orally	Human
Lamiaceae	*Salvia acuminata* Ruiz & Pav.	Anchino (M)	KTG48	Diarrhoea alone	Chewing the leaf	Human
Fabaceae	*Senna* spp.	Diko (M)	KTG84	Joint ache and breakage of bones	Leaf rubbed on affected parts and some leaf chopped and soaked in warm water and drenched	Human and Veterinary
Malvaceae	*Sida* spp.	Moishita (M)	KTG25	Anti-parasitic/ to fatten calf	Leaf chopped and soaked in water and the filtrate is drenched repeatedly	Veterinary
Solanaceae	*Solanum bellum* S. Knapp	Ondod (M)	KTG22	Coughing of equines(Busa)	Root chopped and mixed with cold water and the filtrate drenched either by nose or mouth	Veterinary
	*Solanum acaule* Bitter	Mushta (A)	KTG62	Removes retained placenta	Root chopped and mixed with cold water and the filtrate is applied nasally	Veterinary
	*Solanum acuminatum* Ruiz & Pav.	raki (A)	KTG82	To detach retained placenta	Root chopped, mixed with cold water and drenched orally	Human and Veterinary
	*Solanum incanum* L.	Garint (A)	KTG06	Epizootic lymphangitis(tushita)	Root chopped and mixed with cold water and drenched via nose	Veterinary
	*Solanum* spp.	Danni (M)	KTG50	Poisonous to animals	Root chopped and concoction with water until paste is formed, rubbed on arrow tip & used for hunting	Human and Veterinary
Bignoniaceae	*Stereospermum kunthianum* Cham.	Addi (M)	KTG29	Abdominal pain	Root chopped and mixed with water/coffee and taken in *ad libitum*	Human
Asteraceae	*Tagetes* spp.	Businae (A)	KTG88	Muscle cramp and joint pain	Leaf rubbed well and oils from the leaf are swabbed on areas where pain felt. Fumigation is also possible. Boiled filtrate is drenched	Human
	*Tagetes minuta* L.	Kawato (M)	KTG36	Diarrhea and vomiting	Leaf chopped and ground and the drench the filtrate	Human
Euphorbiaceae	*Tragia doryodes* M.G.Gilbert	Anderta (A)	KTG16	Anthrax	The leaf is chopped and mixed with *Dombya* spp. and the filtrate taken orally	Veterinary
Caryophyllaceae	*Vaccaria hispanica* (Mill.) Rauschert	Sanba tesha (M)	KTG69	For contagious bovine pleuropneumonia and contagious caprine pleuropneumonia	Root chopped and mixed with large amount of water. It gets bloody color (the bloody color indicates appropriate concentration), then drenched	Veterinary
Verbenaceae	*Verbena officinalis* L.	Guni tesha (A)	KTG56	Snake bite/poison	Leaf squeezed by hand and mixed with water and drenched by water	Human and Veterinary
Solanaceae	*Withania somnifera* (L.) Dunal	Buto/wogare (M)	KTG24	Night mare	Roots powdered and children smoked until they cough	Human
Fabaceae	*Zornia apiculata* Milne-Redh.	Medhanit (A)	KTG10	Abdomen ache and vomiting in children	Fresh root chopped and mixed with cold water and drenched	Human
	*Zornia glochidiata* DC.	Halimi (A)	KTG11	Malaria	Root bark is chopped and boiled/concoction with local drinks and boiled coffee leaf	Human
	*Zornia latifolia* Sm*.*	Medihanit (A)	KTG08	Abdominal pain, vomiting	Fresh leaf chopped and mixed to form filtrate	Human

^*^A=Aari; M=Maale; B=Benigna; Amh = Amharic.

This is the first study that documented plants used for disease control by the three ethnic groups in South Ethiopia. Previous studies have documented indigenous knowledge of medicinal plants and medicinal plant practices used in other parts of the country and by other ethnic groups including those in southern Ethiopia [24,25], northern and northwestern Ethiopia [8,26-28], and southwestern Ethiopia [29-31]. Our study thus complements existing studies but also extends them to pastoral areas where the ecology, practices, biodiversity, accessibility and cultural acceptability of medicinal plants are very different from the highlands. The aforementioned reports and our study taken together capture a wide range of different ethnic and social groups, which is a reflection of the richness of knowledge in use of plants for medicinal purposes, and the significance and cultural acceptability of plant based medicinal practice in large parts of Ethiopia. At the same time, this indicates that plant diversity and use of plant based remedies remain decisive for managing human and livestock health in countries like Ethiopia, as is the case for many other countries [7,32-46].

### Ailments treated and ICF

Plants were clustered into 12 different groups based on the use citations by the informants and other end users (Table 1) in order to calculate the ICF. In our study, the ICF values range from 0.72 for evil eye and to 1.00 for rabies. Thus, all clusters had an ICF value greater than 0.5 and hence all of them could be considered for validation of bioactivity and isolation and characterization of the active principles by interested and potential researchers in each cluster.

The highest number of plant species were reported to be used for treatment of abdominal/stomach disorders and internal parasites (22 species, 24.2%), followed by evil eye spirit (11 species, 12.1%), malaria and anemia like syndrome with jaundice, and snake bite/poisoning (10 species, 10.9% each), skin conditions (skin infections and ecto-parasites) and removal of retained placenta (7 species, 7.7% each), coughing in equines and ruminants and pain related illness (5 species, 5.5% each), anthrax (4 species, 4.4%), mich and megagna (an ailment characterized with fever, headache and sweating) and black leg (3 species, 3.3% each) and rabies (1 species, 1.1%) as shown in Table 1.

Animal diseases are one of the major reasons for poor livestock performance in Ethiopia [33], and the use of conventional medicine by smallholder livestock owners is constrained by their high prices and inaccessibility. On the other hand, Ethiopia is characterized by having diverse ecology and diverse mix of socio-cultural and linguistic groups, which might have contributed to the existence of rich knowledge in managing and using large numbers of different medicinal plants against both human and livestock ailments [32]. Therefore, in the absence of use of modern medicine to treat livestock diseases in smallholder livestock production systems, the use of traditional medicinal plants will remain a vital component of Ethiopian livestock production for some years to come. For instance, ethnoveterinary uses of the plant species *Caylusea abyssinica, Cissampelos mucronata, Cissampelos pariera, Desmodium dichotomum, Ipomoea eriocarpa, Justicia diffusa, Premna schimperi,* and *Zornia glochidiata* are reported by these ethnic groups to be effective against selected ecto- and endo-parasites of livestock. Validation of the later through *in vitro* and *in vivo* assessment of their anti-parasitic properties is required to better inform their use by pastoralists and smallholder farmers. Furthermore, bioactivity evaluation of these plant species also help to isolate and purify the active principles by bio-assay guided fractionation for new drug development.

The ICF results could be useful in prioritizing medicinal plants for further scientific validation of plants and plant products [7,8,27,47-49], as pharmacologically effective remedies are expected from plants with higher ICF values [50,51]. Indeed, documentation of inherently rich traditional ethno-medicinal knowledge based on ICF values have provided valuable information on new pharmacological dimensions for better health care of livestock and humans regarding many ailments [50], and also assist conservation and management of rare, gradually vanishing important ethno-medicinal plant species. If validated, the claim for medicinal plants used in traditional medicine for a number of ailments of humans and livestock could provide new applications in supporting health care systems that are urgently needed. In our study, medicinal plant species claimed for anthrax, skin infection and external parasites, pain related illnesses and black leg were cited with the highest ICF values followed by those used to treat coughing in equines and ruminants, malaria and anemia like syndrome with jaundice, abdominal/stomach disorders and internal parasite and retained placenta. The lowest ICF value was recorded for the medicinal plant used to treat evil eye spirit. However, none were below 0.5, which would typically result from plant use to treat rare diseases [27,49], suggesting that our survey addressed medicinal plant species commonly used to treat common human and veterinary ailments in the study areas. Moreover, the highest numbers of plant species were reported to be used for treatment of abdominal/stomach disorders and internal parasites whereas the lowest number of medicinal plant species were reported for the treatment of rabies (Table 1). This implies that stomach disorders and endoparasite infections are likely the more common health problems of human and livestock in the three ethnic groups. Parasite-based health problems in human may be due to domestic hygiene, shared use of water from the same source for themselves and for their livestock, and zoonotic parasite infection. The parasitic health problem in livestock in the study areas could be associated with the ectoparasites particularly ticks and mange mites, increasing the risk for vector born diseases. The internal parasitic health problem in livestock in the study areas are a serious threat during humid season as the condition favors the infection, multiplication and transmission of endoparasites.

### Habits of growth

**Figure 3:**
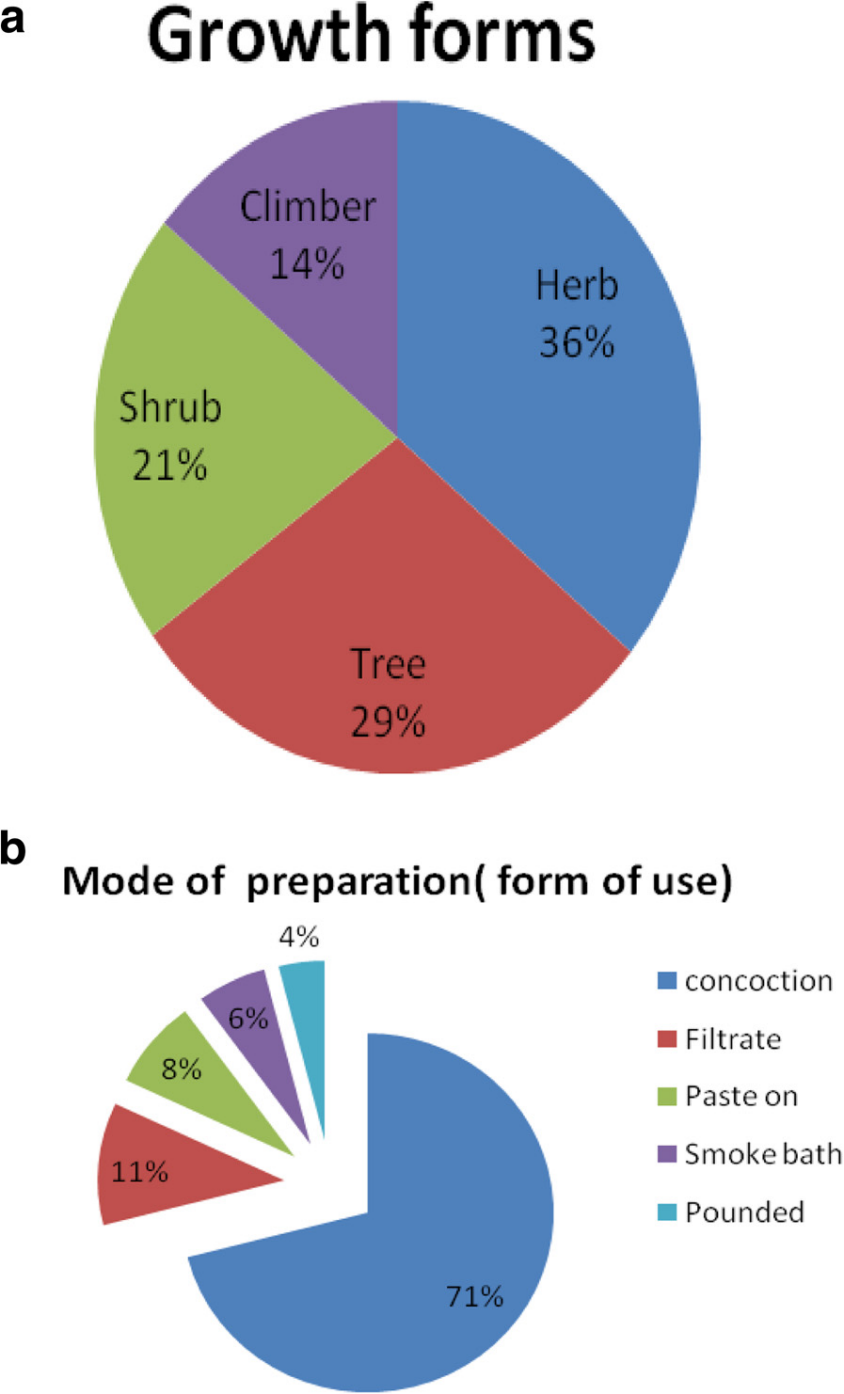
Proportions of growth form (a) and form of use (b) of medicinal plants identified in South Omo for treatment of different human and livestock ailments in South Omo zone, southern Ethiopia.

Figure 3a shows that woody plants made up 50% of the growth form of the plants claimed by the healers for having medicinal properties (29% trees and 21% shrubs), followed by herbs (36%) and climbers (14%). The high proportion of woody plants in our survey is likely associated to the ability of trees and shrubs to withstand long dry seasons, thus resulting in their abundance and year round availability in arid and semi-arid areas. This finding is contrary to the general patterns seen in most medicinal plant inventories where herbs are the largest plant growth forms [23,25,27,53]. A high usage of herbs in some studies could be an indication of their abundance, especially in areas receiving year round rainfall. Thus, the variation in parts of medicinal plants used may be related to differences in seasonality though also arise from differences in socio-cultural beliefs, and practices of the healers of different regions or countries.

### Mode of preparation (form of use)

Concoction, filtrate (a liquid from which insoluble impurities have been removed), paste on (topical), pounded and smoke bath are common use forms or modes of preparations reported in our study, with concoction (71%) and filtrate (11%) as the major use forms of the plants cited (Figure 3b). The remedies are prepared using water (hot or warm), local drinks, boiled coffee or milk as a carrier and taken either orally or through inhalation of the vapor after boiling (smoke bath treatment). Within the total number of claimed medicinal plants, healers used 14 plants (15.4%) by mixing of two plants to treat selected ailments. For instance, *Geranium arabicum* mixed with *Laggera tomentosa* is used for the treatment of epizootic lymphangitis in animals; *Droguetia iners* mixed with *Premna oligotricha* is used for the treatment of malaria in humans, and *Dombeya* spp. mixed with *Tragia doryodes* is used for the treatment of anthrax. The frequent use of concoction and the mixing of two or more plants by healers could be associated with healer’s belief of synergistic effects of certain plant components for healing the illnesses. This finding is consistent with earlier reports [26,54,55] but disagrees with other studies where crushing and squeezing [27,28] and homogenizing and crushing [24] were the main use forms. It is likely that these differences are associated with the differences in culture and knowledge in different socio-cultural groups.

### Parts of plant used

**Figure 4:**
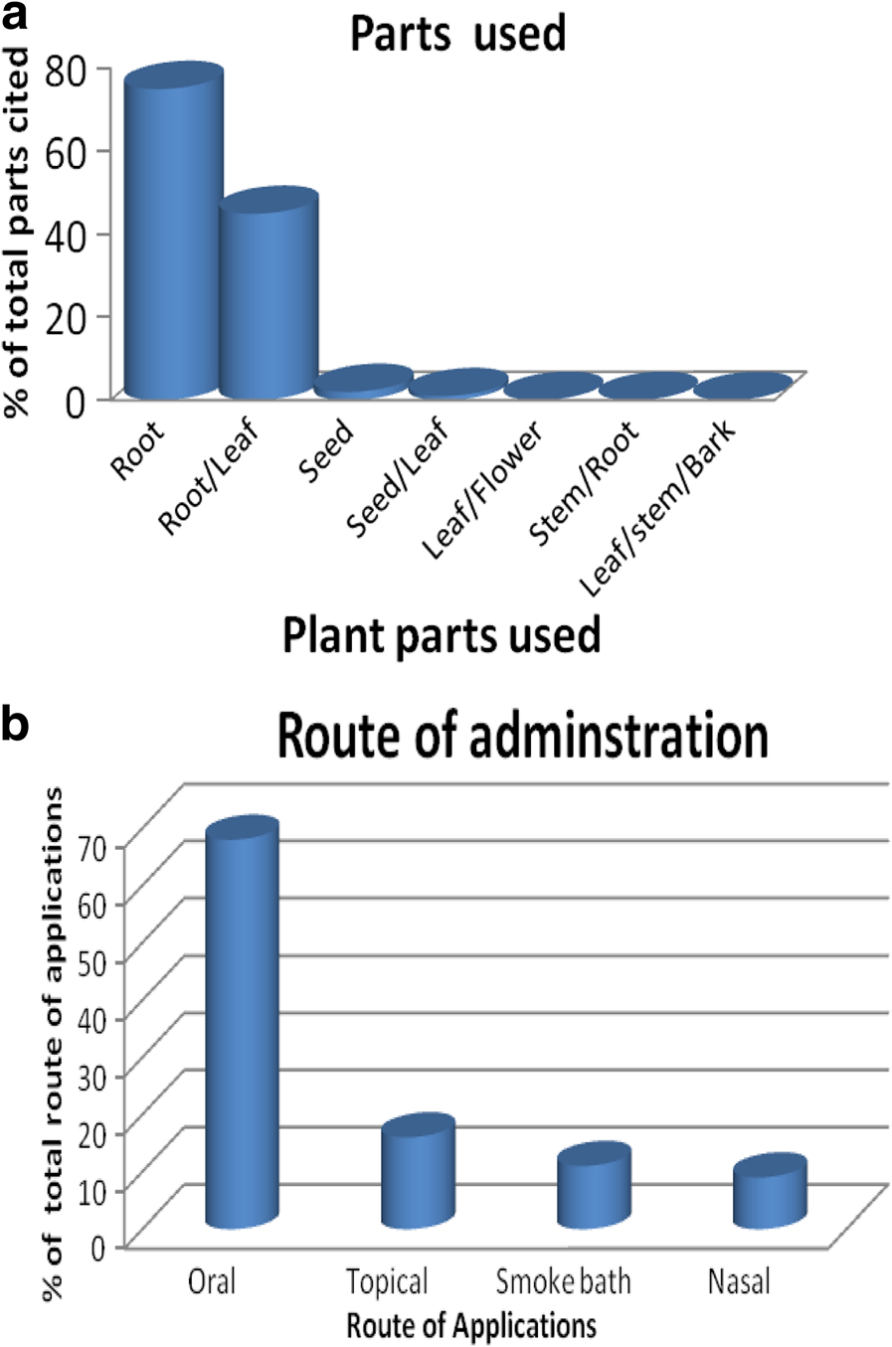
Proportion of plant parts used for medicinal purposes (a) and route of administration of plant preparations (b) for treatment of human and livestock ailments in South Omo zone, southern Ethiopia.

Almost all plant parts, including roots, leaves, stem, bark, fruits, young shoots and flowers, were cited for use in preparing the different remedies. However, roots followed by leaves represented the most common parts used (Figure 4a) for treating ailments in humans and livestock, respectively. Roots appeared to be the main plant part commonly used by the healers in the current study area. This could be associated with the fact that roots remain in the soil and are easily available, even during the long dry seasons in arid and semi-arid areas. In addition, the use of plants root could also be associated with early African beliefs in their powerful therapeutic effects. For example, early African diasporas in the Americas and those migrants to Caribbean countries during the colonial period used plant roots to protect against malaria and venereal diseases and to induce abortions, but also to prepare favorite household alcoholic drinks, as roots contributed to alcohol fermentation, color, flavor, and foam formation [20,56,57]. However, the use of medicinal plant roots, either for immediate use of treating ailments or for commercialization purpose to generate income, could also negatively contribute to local biological diversity and conservation because of complete plant removal from its natural habitat. The common use of leaf in the preparation of remedies could partly be due to the relative ease of finding this plant part. In agreement with our study, similar studies in other parts of Ethiopia reported that roots and leaves are indeed the most commonly used medicinal plant parts [27,28].

### Routes of administration and dosage used

Both internal and external applications were reported by the informants in the treatment of various human and livestock ailments in our study. The commonly reported routes of administration are oral (65%), followed by topical (15%), nasal (10%) and smoke bath treatment (10%; Figure 4b). The choice of oral administration may be related to the use of some solvents or additives (milk, butter, alcoholic drinks, boiled coffee, and food) that are commonly believed to serve as a vehicle to transport the remedies. The additives are also important to minimize discomfort, improve the taste and reduce adverse effects such as vomiting and diarrhoea, and enhance the efficacy and healing conditions [31]. Similar findings were reported by many other researchers, indicating the oral route as the most preferred mode of administration [25,28,58-64]. However, there is no consensus on the dosage used and frequency of the medication among healers. For example, the dosage varied according to the type of illness ranging from two spoonfuls (e.g. for treatment of anemia like syndrome with jaundice using concoct prepared from *Cissampelos* spp*.*) to a cup or glass full (e.g. for treating “busino” or abdominal pain using decoct from *Centella asiatica).*

## Conclusion

This study showed that traditional medicine, mainly involving the use of medicinal plants, is playing a significant role in meeting the primary healthcare needs of the three ethnic groups. Acceptance of traditional medicine and limited access to modern healthcare facilities could be considered as the main factors for the continuation of the practice.

This field survey has documented 91 plant species distributed across 33 families and 57 genera as having medicinal properties against 34 human and livestock ailments as reported by healers from Aari, Maale and Bena-Tsemay ethnic groups, complementing previous studies from other ethnic groups in Ethiopia. The highest number of plant species was reported to be used for treatment of abdominal/stomach disorders and internal parasites. Woody plants (trees and shrubs) were the main form used, likely related to the long dry seasons typically occurring in the residential area of the ethnic groups studied. Concoction appeared to be the most popular use form in the current study. The most commonly used route of administration is oral. This study contributes to the enormous indigenous knowledge on medicinal plants and plant-based remedies practiced among ethnic groups, and it assists knowledge and practice preservation, which remain mostly with elderly traditional practitioners. Furthermore, the information generated will also inform future validation studies, so as to increase the acceptability of plant-based remedies in human and animal health care systems both nationally and internationally.

## Competing interests

The authors declare that they have no competing interests.

## Authors’ contributions

KT, ED and AT carried out field survey and data analysis, ED and KT prepared the initial structure of the draft manuscript and KT, ED and AT revised the manuscript critically to the present form. GG introduced us to the people in the study and was involved in a preliminary survey. SA and JH secured funding for the project, assisted data interpretation, manuscript structuring and provided input to previous drafts resulting in the present form. All authors read the final manuscript and agreed on its submission.
